# Efficacy and safety of apatinib in patients with previously treated metastatic colorectal cancer: a real-world retrospective study

**DOI:** 10.1038/s41598-018-22302-z

**Published:** 2018-03-15

**Authors:** Miaomiao Gou, Haiyan Si, Yong Zhang, Niansong Qian, Zhikuan Wang, Weiwei Shi, Guanghai Dai

**Affiliations:** Chinese PLA General Hospital, Oncology Department, Beijing, China

## Abstract

No definitive treatment strategy has been established for patients with metastatic colorectal cancer (mCRC) who experienced progression after three or more lines of chemotherapy. A total of 36 mCRC patients were enrolled in this retrospective study who received apatinib therapy under non-clinical trial setting after progression in People’s liberation army general Hospital from March 2015 and August 2017. Progression free survival (PFS), overall survival (OS), disease control rate (DCR), objective response rate (ORR) and treatment-related adverse events (AEs) were reviewed and evaluated. Five patients achieved partial response (PR), and 25 achieved stable disease (SD), and 6 achieved progression disease (PD), illustrating a DCR of 83.3% and an ORR of 13.9%. Median PFS was 3.82 m and median OS was not reached. The toxicities associated with apatinib were generally acceptable with a total grade 3/4 adverse event incidence of 27.8%. The most common grade 3/4 adverse events were hypertension (n = 4, 11.1%), liver function damage (n = 3, 8.3%) and hand–foot syndrome (n = 2, 5.6%). No drug-related death occurred. Apatinib therapy provides a reasonable option with an acceptable safety profile for Chinese mCRC patients failed to prior chemotherapy.

## Introduction

Metastatic colorectal cancer (mCRC) is one of the most prevalent malignancies in China, which poses a great threat to human health^1^. For patients with mCRC, fluorouracil combined with either oxaliplatin or irinotecan are recommended as first and second line treatment and biological agents targeting vascular endothelial growth factor (VEGF) and epidermal growth factor receptor (EGFR) are also available, such as bevacizumab and cetuximab^2,3^. No standard treatments have been accepted in third line treatment so far. But some studies have made different attempts and showed different results, involving anti-EGFR, anti-PD-1 and anti-angiogenesis strategies. Panitumumab plus chemotherapy was proved to prolong the progression free survival (PFS) and overall survival (OS) compared with chemotherapy alone^4^. Programmed cell death-1 (PD-1) blockade has showed higher efficacy in tumors with mismatch-repair deficiency^5^.

Anti-angiogenesis is an important anti-cancer strategy^6^. The VEGF inhibitor tends to prolong PFS and OS in CALGB 80405 and Fire-3 study^2,7^. The tyrosine kinase inhibitors (TKIs) targeting VEGF receptor such as sunitinib and sorafenib performed only limited clinical benefits in mCRC^8,9^. Regorafenib, an oral small-molecule multi-kinase inhibitor targeting signaling pathways including VEGFR1-3 significantly prolonged median PFS (3.2months) compared with placebo in phase III trial^10^.

Recently, apatinib, a novel TKI targeting VEGFR-2, has shown promising efficacy on various types of cancers with acceptable toxicities^11–14^. In the phase III trial, Li *et al*. showed that apatinib treatment significantly improved OS and PFS in patients with advanced gastric cancer^14^. However, no clinical studies with detailed data have investigated the efficacy of apatinib in mCRC after standard treatment. Herein, we conducted a retrospective evaluation of the efficacy and toxicity of apatinib in mCRC after failure of prior treatment and explored the impact of previous anti-antigenic treatment of bevacizumab on the efficacy of apatinib, which derived from the clinical experience of Chinese PLA General Hospital (PLAGH).

## Materials and Methods

### Patient eligibility

This is a retrospective real world study. This study has been approved by the ethics committee of Chinese PLA General Hospital. Informed consent was reviewed and signed by the patients or their legal guardian.

Patients with advanced or metastatic colorectal cancer who had progressed or relapsed after undergoing at least two lines of systemic therapy in accordance with the recommendations and guidelines of NCCN (National Comprehensive Cancer Network) in our center were included. The inclusion criteria were shown as follows: (1) patients with histologically confirmed colorectal cancer, who were treated with apatinib in any line from March 2015 and August 2017 in PLAGH were included; (2) patients with at least one measurable lesion, which was defined by Response Evaluation Criteria in Solid Tumors (RESIST) criteria (1.1) and had done at least one measurement. For patients who have been treated with bevacizumab, there were no limits regarding the time since last treatment of bevacizumab before apatinib treatment. Data were retrospectively obtained from patients’ medical history.

### Treatment and dose modification

Apatinib was initially administered from the dose of 250 mg once daily and in some instances was added to 500 mg according to patients’ tolerance and request. The treatment could be interrupted, reduced to 125 mg or elevated to 850 mg once daily, or permanently discontinued due to its severe adverse events. The apatinib combined with other agents was up to doctors’ choice and patients’ general performance status. One treatment cycle was based on regimens either 21 days or 28 days. Patients were followed up till disease progression, death, discontinuation of treatment due to intolerable toxicity, or till the cutoff date of August 8, 2017.

Only patients who had finished at least one cycle apatinib therapy and evaluated the efficacy were included in this study.

### Efficacy and safety assessments

Primary analysis endpoint was progression-free survival (PFS) and secondary analysis endpoint was disease control rate (DCR), objective response rate (ORR) and overall survival (OS).

PFS was defined as the time period from initiating apatinib treatment to disease progression or death, whichever came first. OS was defined as the time period from initiating apatinib treatment to the date of death of any cause or last follow-up visit.

Tumor responses were assessed by both radiologists and oncologists every two cycles or significant signs of progression appeared or necessary. Objective tumor responses were assessed according to RECIST criteria (1.1). Tumor responses included complete response (CR), partial response (PR), stable disease (SD), and progressive disease (PD). DCR was defined as the addition of objective response and stabilization rates (CR + PR + SD). ORR was defined as the addition of CR and PR. Toxicities were reviewed and determined from patients’ medical history and laboratory examination results or from telephone follow-up according to the National Cancer Institute Common Toxicity Criteria for Adverse Events version 4.0 (CTC4.0).

### Statistical analysis

Quantitative data are presented as median (range) or number of patients (percentage). Survival analysis was conducted by the Kaplan–Meier method and compared by the log-rank test. Exploratory univariate analyses were performed with the log-rank test using the following variables: age, gender, location, KRAS status, line of apatinib, and combinational treatment and bevacizumab prior to apatinib. Cox multivariate models were performed based on the univariate analyses results. AEs were summarized using percentages and frequency counts.

Statistical analysis was performed using SPSS version 18.0 (SPSS Inc., Chicago, IL, USA). P < 0.05 was regarded as statistically significant.

## Results

### Patient demographics

Ninety-three patients with mCRC were prescribed with apatinib between March 2015 and August 2017. Fifty-seven patients were excluded because of lack of follow-up medical data. Consequently, a total of 36 metastatic colorectal cancer patients were included in the final analysis. Clinic pathological characteristics at the initiation of apatinib were shown in Table 1. Twenty-nine patients took 250 mg and seven patients took 500 mg at the beginning.

**Table 1:** Clinical characteristics of the study population (n = 36).

Characteristics	No. (%)
Age (median)	
<60	25(69.4)
≥60	11(30.6)
Gender	
Male	17(47.2)
Female	19(25.8)
ECOG performance status	
0–1	25(69.4)
≥2	11(30.6)
Location	
Left	28(77.8)
Right	8(22.2)
KRAS status	
unknown	7(19.4)
wild	17(47.2)
mutant	12(33.3)
Line of apatinib	
3 line	21(58.3)
Further line	15(41.7)
Apatinib combined	
Yes	22(61.1)
No	14(38.9)
Bevacizumab prior to apatinib	
Yes	23(63.9)
No	13(36.1)

ECOG: Eastern Cooperative Oncology Group, Location: splenic flexure, descending colon, sigmoid colon, or rectum were classified as left sided, appendix, cecum, ascending colon, hepatic flexure, or transverse colon were classified as right-sided mCRC.

### Efficacy

**Figure 1:**
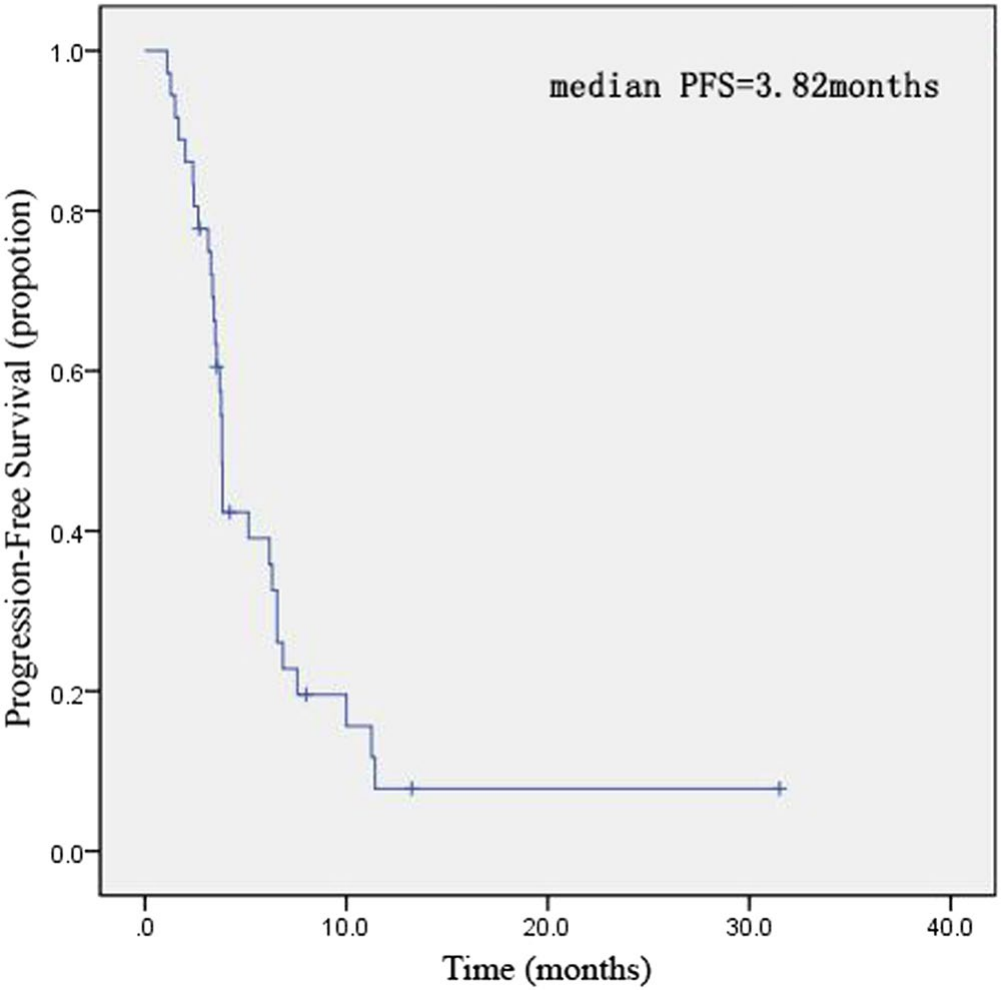
Kaplan–Meier estimates of progression-free survival of metastatic colorectal patients received apatinib treatment.

At the end of follow up, median PFS was 3.82 months (95% CI: 3.664–3.979). Six patients are currently taking apatinib with no progression. Twelve (33.3%) patients died and overall survival data were not achieved at the time of the analysis. The Kaplan–Meier analysis of PFS was shown in Fig. 1.

Among 36 patients with evaluable best response, 5 patients (13.9%) achieved PR, 25 patients (69.4%) had SD, and 6 patients (16.7%) reported PD, but CR was not achieved in all the patients. This resulted in a DCR of 83.3% and an ORR of 13.9% in Table 2.

**Table 2:** Tumor response.

Response (n = 36)	No. (%)
CR	0(0)
PR	5(13.9)
SD	25(69.4)
PD	6(16.7)
ORR	5(13.9)
DCR	27(83.3)

CR: complete response; PR: partial response; SD: stable disease;

PD: progression disease; ORR: CR + PR; DCR: CR + PR + SD.

As shown in Table 3, univariate analysis indicated that there was no significant association of PFS with gender (P = 0.358), age (P = 0.414), line of therapy (P = 0.520), location (P = 0.619) and ECOG performance status (P = 0.078). The KRAS mutation status were statistically associated with PFS of apatinib treatment (P = 0.627). Inter-group analysis found no significant differences in PFS between patients previously treated with or without bevacizumab (P = 0.538). Multivariate analysis showed that factors including gender, line of therapy, location, ECOG performance status, KRAS mutation status had no significant association with PFS.

**Table 3:** Log-rank analysis of factors for PFS.

Variable	No. of cases	PFS (median, 95% CI)		*P*-value
Total patients	36	3.82	(3.664–3.979)	
Median Age range				
<60	25	3.86	(0.078–7.636)	0.069
≥60	11	3.36	(2.327–4.387)	
Gender				
Male	17	5.14	(0.657–9.629)	0.358
Female	19	3.82	(3.470–4.173)	
ECOG performance status				
0–1	25	6.2	(2.441–9.917)	0.078
≥2	11	3.57	(3.318–3.825)	
Location				
Left	28	3.82	(2.012–5.631)	0.619
Right	8	3.86	(3.430–4.284)	
KRAS status				
unknown	7	3.86	(2.267–5.448)	0.627
wild	17	3.71	(3.330–4.098)	
mutant	12	3.82	(3.664–3.979)	
Line of apatinib				
3 line	21	3.82	(3.668–3.975)	0.52
Further line	15	5.14	(2.578–7.707)	
Apatinib combined				
Yes	22	5.14	(1.878–8.408)	0.414
No	14	3.71	(3.190–4.238)	
Bevacizumab prior to apatinib				
Yes	23	3.79	(1.491–6.224)	0.538
No	13	3.86	(3.387–4.185)	
Hypertension				
Yes	10	6.90	(0.000–15.577)	0.055
No	26	3.54	(3.100–3.900)	
Hand-food syndrome(HFS)				
Yes	12	3.83	(1.876–5.724)	0.347
No	24	3.82	(3.476–4.124)	
Nausea and Vomiting				
Yes	12	3.80	(3.263–9.337)	0.674
No	24	6.30	(3.339–4.251)	

Univariate analysis indicated that there was no significant association of PFS with age,gender, ECOG PS, location, KRAS status, line of therapy, Apatinib combined or not and Bevacizumab prior to apatinib. p values by log-rank test are displayed.

### Safety

All patients were included in the safety analysis set. Toxicities were generally well tolerated. All toxicities occurring in the patients are shown in Table 4. 27.8% of the patients developed grade 3 adverse events. Grade 4 toxicity was not observed in all the patients. Hand-foot syndrome, nausea and vomiting, hypertension were the top 3 adverse events in this study. The most common grade 3 adverse events were hypertension (n = 4), liver damage (n = 3) and hand–foot syndrome (n = 2). In most cases, the hypertension was mild and controllable by oral hypertension agent. One patient discontinued the apatinib treatment because he developed proteinuria. Two patients withdraw therapy due to own reason and enrolled in other clinical trial. Seven patients required dosage reductions.

**Table 4:** Analysis of adverse event.

Adverse Event	No. (%)	
	Any Grade	Grade 3 or 4
Non-hematologic		
Hand-foot syndrome	12(33.3)	2(5.6)
Nausea,Vomiting	12(33.3)	0
Hypertension	10(27.8)	4(11.1)
Diarrhea	7(19.4)	1(2.8)
Liver damage	6(16.7)	3(8.3)
Fatigue	5(13.9)	0
Proteinuria	5(13.9)	1(2.8)
Myelosuppression	5(13.9)	0
Gastrointestinal bleeding	4(11.1)	0
Rash	4(11.1)	0
Canker sores	2(5.6)	0
Elevated transaminase	2(5.6)	0
Hoarseness	2(5.6)	0
Hematologic		
Neutropenia	4(11.1)	1(2.8)
Leukopenia	2(5.6)	0
Thrombocytopenia	1(2.8)	0

Based on the top 3 adverse events in this study, we made try to analysis whether hypertension, hand-foot syndrome, nausea and vomiting were associated with PFS, finding no significant association (p = 0.055, p = 0.347, p = 0.674).

## Discussion

With regard to present study, the therapeutic strategies of mCRC have stepped into targeted era. The combination of molecular targeted agents and chemotherapy has been recommended for mCRC patients in NCCN guideline as standard first and second line therapy^2,4^. The standard third line therapy of mCRC hasn’t been established. In this study, we assessed the efficacy and safety of apatinib in mCRC patients and explore the impact of previous anti-angiogenic treatment of bevacizumab on apatinib. In addition, we also reported an adverse event of hoarseness, which has not been documented in the instructions.

Apatinib, also known as YN968D1, is a novel oral small-molecule TKI selectively targeting VEGFR-2, which binds all VEGF-A isoforms, VEGF-C, and VEGF-D^15^. The blockage of signaling pathways of VEGF-A to VEGFR-2 can inhibit the cellular proliferation, migration and endothelial cell survival^16^. Several studies have confirmed that blocking VEGFR-2 is a promising therapy for inhibiting angiogenesis^17^. Apatinib has been demonstrated in several types of cancers. One study reported apatinib in advanced breast carcinoma patients who failed standard treatment (PFS = 4.90 m, OS = 10.3 m)^18^. Apatinib was also reported to be an effective salvage therapy agent in lung cancer with PFS of 4.6 months and OS of 6.0 months^19^. A retrospective study reviewed the efficacy and safety of apatinib in stage IV sarcoma with median PFS of 8.84 months^20^. In the phase III trial, apatinib has been shown benefit patients’ survival compared with placebo in advanced gastric cancer (6.5 vs. 4.7 months, P = 0.0149) and has been approved by Chinese Food and Drug Administration (CFDA) for the treatment of patients with advanced gastric cancer after two or more lines of prior chemotherapy^14^. Several cases treated with apatinib published in PubMed have shown promising efficacy^21–23^. However, there is still controversy over its efficacy and safety and the impact of previous anti-antigenic treatment remains unknown^24,25^.

Hereby, in this single-center retrospective study, we reported the efficacy and safety of apatinib in mCRC. Median PFS in our study was of 3.82 m, which seemed to be comparable with regorafenib reported in previous studies. In CONCUR study, 204 patients were assigned to receive either regorafenib (n = 136, 67%) or placebo (n = 68, 33%), and regorafenib significantly prolong the mPFS and mOS compared with placebo (PFS: 3.2 m vs. 1.7 m, HR = 0.31, 95% CI: 0.22–0.34, P < 0.001; OS: 8.8 m vs. 6.3 m, HR = 0.55, 95% CI: 0.395–0.765, P = 0.0002). DCR in CONCUR study was 51%, which was 7 times higher than placebo group^10^. The mPFS of apatinib is numerically longer, suggesting a promising therapeutic effect. In another randomized controlled trial, addition of aflibercept to FOLFIRI regimen improved PFS (PFS: 6.90 m vs. 4.67 m, HR = 0.758, 95% CI: 0.661–0.869, P = 0.001)^26^. PFS of apatinib in our study seems shorter than this study. The potential reasons are as follows: first, apatinib in real-world practice was used in later line compared with aflibercept in clinic trial in second line; second, regimens in our study was apatinib alone or combined with only one mild chemotherapeutic agent while aflibercept combined with FOLFIRI in this study, which was regard as intensive regimens usually. These discrepancies highlight the gap between randomized controlled trials and the real-world practice. The TERRA Study showed us that Trifluridine/tipiracil has a statistically significant survival benefit compared with placebo in Asian patients with mCRC refractory or intolerant to standard chemotherapies, regardless of exposure to biologic therapy^27^. Median overall survival was significantly longer in the Trifluridine/tipiracil than in the placebo arm (7.8 months [95% CI, 7.1 to 8.8 months] v 7.1 months [95% CI, 5.9 to 8.2 months], respectively. It also provides an option for Asian patients with previously treated mCRC. However, aflibercept and regorafenib and Trifluridine/tipiracil have not been accessible in Chinese mainland, even not to mention the Chinese medical care. In our study, mOS could not be calculated, because only 12 patients had reached death events till the cut-off date. A longer follow-up time for the cohort would be helpful for data collection and calculation.

Our study revealed 5 PR, 25 SD and 6 PD patients according to RECIST criteria. The DCR was 83.3% and ORR was 13.9%, which are consistent with those reported in breast cancer (DCR: 68.9%, ORR: 22.2%), non-small cell lung cancer (DCR: 61.9%, ORR: 9.5%) and sarcomas (DCR: 80%, ORR: 20%)^18–20^. DCR of regorafenib was 51%, which indicated apatinib had efficacy as third or further line compared with regorafenib based on a cohort with more patients having a ECOG PS < 2^10^.

Neither univariate analysis nor multivariate analysis using the Cox model illustrated significant association of PFS with age, gender, ECOG PS, location and line of apatinib, which was similar to the results in gastric cancer treated with apatinib in phase III trial of gastric cancer^14^. Besides that, KRAS status and bevacizumab prior to apatinib were also not statistically associated with PFS after apatinib treatment. The reason may be lack of big sample data. Up to now, there is no direct evidence proving that previous exposure of bevacizumab benefit apatinib as second anti-antigenic agent in colorectal cancer. Only a phase I trial investigating the efficacy of tivozanib in metastatic breast cancer showed that four patients who previously exposed to bevacizumab achieved partial response^28^. Above all, previous anti-antigenic treatment seems not to be a contraindication of apatinib treatment. Further studies are needed to evaluate how long the washout period will take before the administration of apatinib as second line anti-antigenic treatment.

Regarding to the safety of anti-angiogenic agents, hypertension, hand-foot syndrome and proteinuria are the most common adverse events of as reported previously in clinical trials^29^. In phase III trial of gastric cancer, grade 3/4 hypertension, proteinuria and hand-foot syndrome occurred in 11.1%, 2.8% and 5.6% of patients respectively in the apatinib group^14^. In our study, the most common grade 3 adverse events were hypertension (11.1%), liver damage (8.3%), and hand-foot syndrome (5.6%). The toxicities of apatinib are similar to or better than those of other TKIs such as sorafenib and sunitinib^30,31^. In the present study, we involved the dosage of 250 mg once daily or in some instances 500 mg once daily, and the results showed that less than 1/3 of these patients developed grade 3 AEs, and nobody showed grade 4 AEs. Furthermore, common hematologic toxicities related to apatinib included neutropenia (11.1%) as compared with 5.3% in gastric trial. The incidence of hematologic toxicities was a little higher mainly because of the combination with toxic chemotherapy. Although most of the toxicities were well tolerated, many patients still experienced interruption, dose reduction and discontinuation during treatment.

Encouragingly, one patient managed to escalate to 850 mg per day with hand–foot syndrome (grade 2), diarrhea (grade 1) and rash (grade 1), indicating that patients’ tolerance to dosage and toxicity was individually different. Recently, it has been suggested that the occurrence of specific adverse events, such as hypertension, hand-foot syndrome and proteinuria during anti-antigenic therapy might be associated with improved efficacy^32^. We had tried to perform PFS and OS analysis based on the adverse events and found no significant association between PFS and AEs. This phenomenon had not been observed in this study.

However, this study was subject to limitions of its retrospective observational methodology, including potential missing data, possible information bias, small size and lacking of control group. Quality of life was not assessed. We just showed the real-world date, which need to be validated in multi-center randomized controlled double-blind clinical trials and further follow-up.

## Conclusion

As a single-center retrospective study with limited sample size, our study explore the apatinib used in metastatic colorectal cancer. As we see from study, apatinib has encouraging efficacy and safety and provides an option with an acceptable safety profile for mCRC patients who were refractory to two or more lines of prior chemotherapy.
